# Seasonal influenza vaccine and frequency of adverse events in health workers receiving two doses of AstraZeneca vaccine: a retrospective cohort study

**DOI:** 10.1016/j.virusres.2026.199757

**Published:** 2026-05-29

**Authors:** Masoud Sedaghat, Farid Najafi, Fatemeh Khosravi Shademani, Shahab Rezaeian

**Affiliations:** a Student Research Committee, School of Public Health, Kermanshah University of Medical Sciences, Kermanshah, Iran; b Research Center for Environmental Determinants of Health, Health Institute, Kermanshah University of Medical Sciences, Kermanshah, Iran; c Social Determinants of Health Research Center, Social Health Research Institute, University of Social Welfare and Rehabilitation Sciences, Tehran, Iran; d Infectious Diseases Research Center, Health Policy and Promotion Institute, Kermanshah University of Medical Sciences, Kermanshah, Iran; e Dept. of Epidemiology, School of Health, Kermanshah University of Medical Sciences, Kermanshah, Iran

**Keywords:** Seasonal influenza vaccine, AstraZeneca vaccine, Relative risk reduction, Number needed to vaccinate, Retrospective cohort study

## Abstract

**Background:**

Previous studies suggest that co-administration of COVID-19 and influenza vaccines is safe and maintains immunogenicity. However, evidence on the association between prior seasonal influenza vaccination and adverse events following COVID-19 vaccination remains limited. This study evaluated the relationship between prior influenza vaccination and adverse events after the second dose of the AstraZeneca COVID-19 vaccine among healthcare workers (HWs).

**Methods:**

In this retrospective cohort study, 780 HWs who received two doses of the AstraZeneca vaccine were included. The outcomes were the occurrence and frequency of post-vaccination adverse events. Generalized linear models with binomial distribution and log link function were used to estimate adjusted risk ratios (RRs) for individual adverse events. Poisson regression models estimated adjusted incidence rate ratios (IRRs) for the number of reported adverse events. Relative risk reduction (RRR) and number needed to vaccinate (NNV) were also calculated for selected outcomes.

**Results:**

Overall, 95.5% of participants reported at least one adverse event after vaccination, with three adverse events being the most common frequency (n = 210). Prior influenza vaccination was associated with a lower frequency of adverse events (adjusted IRR=0.83, 95% CI: 0.76–0.91). Older age (>40 years) and male sex were also associated with fewer reported adverse events. Influenza vaccination significantly reduced the risks of fever, chills, body pain, gastrointestinal symptoms, and fatigue. Subgroup analyses by age and sex showed generally consistent findings.

**Conclusion:**

Prior influenza vaccination was associated with fewer adverse events following AstraZeneca vaccination. Older age and male sex were also linked to lower frequencies of reported adverse events.

## Highlights

• Body pain (71.5%) and fatigue (64.6%) were the most common adverse events after AstraZeneca vaccination.
• Prior influenza vaccination was associated with fewer adverse events following AstraZeneca vaccination.
• Influenza vaccination significantly reduced the risks of fever, chills, body pain, gastrointestinal symptoms, and fatigue.
• Gastrointestinal symptoms showed the greatest reduction among influenza-vaccinated participants (RRR=63%).
• The protective association of influenza vaccination was consistent across age and sex groups despite higher adverse-event rates in younger adults and women.

## 1 Introduction

The prevalence of acute upper respiratory infection, abbreviated as Covid-19, emerged in China in December 2019 and spread rapidly in other countries as well (Velavan and Meyer, 2020). This infectious virus caused global public health concern worldwide, so that the World Health Organization (WHO) declared a pandemic status of this disease on March 11, 2020 (Saghazadeh and Rezaei, 2020). Although the lethality rate of Covid-19 has been significantly lower than the SARS and MERS epidemics, its transmission and contagion power is much higher than previous viruses. In addition, considering severe health outcomes and long-term effect of COVID-19, it can be a serious threat for public health in the world (Guan et al., 2020).

In response to an epidemic of an emerging disease, extensive global research will be undertaken to create and produce vaccines. For instance, during the COVID-19 pandemic, over 200 vaccines were developed (Pormohammad et al., 2021). In the clinical trials of the Covid-19 vaccine, several side effects have been reported after the injection, which ranged from mild and local symptoms including pain in the injection area, redness, myalgia, fatigue and fever to rare complications such as anaphylactic shock (Waheed et al., 2021). After the injection of covid-19 vaccines, a person can show a range of mild to severe symptoms of the disease (Ramasamy et al., 2021). Considering that the covid-19 disease is an emerging disease and the vaccination against the disease have been injected into people for the first time, accordingly, investigating the side effects of these vaccines in different populations can help policy-makers to better understand the preventive ways based on the at risk groups.

Studies have shown that following the pandemic of the Covid-19 disease, the injection of the seasonal influenza vaccine can be effective in reducing the incidence, death or alleviating the symptoms of the Covid-19 disease (Marín-Hernández et al., 2021; Noale et al., 2020; Ragni et al., 2020). On the other side, concomitant administration of Covid-19 vaccine and influenza vaccine increases no safety concerns and maintains the antibody response to both vaccine (Lazarus et al., 2021). But limited published study was found regarding the side effects of the Covid-19 vaccine in relation with receiving seasonal influenza vaccine (Rzymski et al., 2022). Therefore, considering the persistence of influenza and covid-19 disease due to the mutation and successive changes of their pathogenic agent and the need to inject the vaccine of these two diseases in the form of reminder doses, the purpose of this study is to determine the relationship between receiving the seasonal influenza vaccine and the occurrence and frequency of side effects of the AstraZeneca vaccine among Healthcare Workers (HWs).

## 2 Methods

### 2.1 Study design

This study was a retrospective cohort study conducted in 2022. The target population was the HWs of Imam Reza and Imam Khomeini hospitals in Kermanshah city, western Iran. In this study, the personnel of the mentioned hospitals who had a history of seasonal influenza vaccination from 1st to 20th October 2020 were considered as exposure group and those without history of receiving seasonal influenza vaccine were considered as non-exposure group.

### 2.2 Sample size and sampling method

**Fig. 1:**
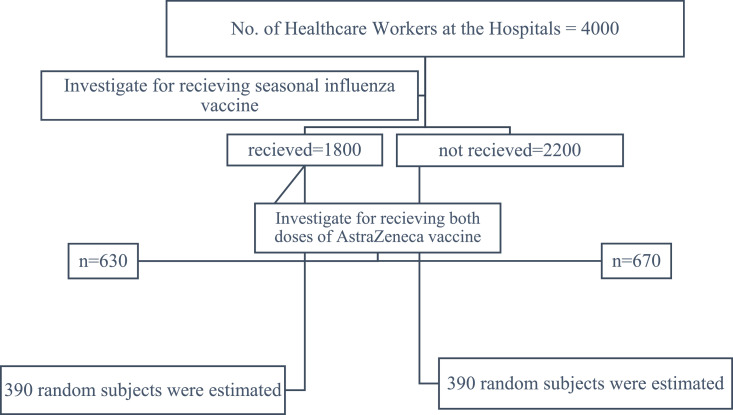
The flowchart of the study.

The calculation of sample size was based on a similar study by Debisarun et al. (Debisarun et al., 2020). Considering the prevalence of complications to be 42% in the exposure group and 54% in the non-exposure group, with a confidence level of 95% and power 90%, the sample size was estimated at 352 subjects for each group. Ten percent additional sample was added to the sample size because of attrition or non-responding of the sample during the study and 390 subjects were then considered for each group. The name list of the HWs at the mentioned hospitals, which included 4000 people, was obtained from the health center of Kermanshah city. After that, among the name list, subjects were stratified based on the receiving or not seasonal influenza vaccination (Fig. 1).

### 2.3 Data source and variables

The information on receiving or not receiving the seasonal influenza vaccine was extracted from the documents of the hospitals and deputy of health of Kermanshah University of Medical Sciences. Then people from these groups (receiving or not the seasonal influenza vaccine) who had received both first and second doses of AstraZeneca vaccine (ChAdOx1) were randomly selected based on the estimated sample size. These HWs included administrative personnel, doctors, medical students and nurses. Demographic and clinical information including age, sex, education, working ward, marital status, underlying disease (have/don't have), and the work experience (in years) as well as the complications of the vaccine were obtained using the researcher-made questionnaire. In order to complete the questionnaires, after checking the inclusion criteria the questionnaires were completed by subjects in the workplace. The inclusion criteria included receiving both the first and second doses of AstraZeneca vaccine, and the exclusion criteria included having a specific or incurable disease and not consenting to participate in the research.

### 2.4 Outcomes

The primary outcome of interest in this study was the side effects of the second dose of AstraZeneca vaccine, which include fever, chills, sore throat, headache, body pain, shortness of breath, runny nose, loss of smell, burning and redness of the eyes, nausea, gastrointestinal symptoms, and fatigue. The duration of the investigation of the side effects was considered to be a maximum of one month after the injection of the vaccine. The information of side effect was collected as yes/no question. In Iran, the AstraZeneca vaccination began in July 2021 and electronic vaccine certificate was mandatory for work.

Also, the frequency of side effects of AstraZeneca vaccine following the administration of second dose of the COVID-19 vaccine was calculated by summing the number of adverse events.

### 2.5 Analysis

In descriptive analysis, frequency (percentage) was used for qualitative variables and mean (standard deviation) for quantitative variables according to receiving or not receiving influenza vaccine. The comparison of the basic characteristics of the two groups that received influenza vaccine or not was done using chi-square test for grouped variables and independent t-test for quantitative variables.

Each adverse event following the second dose of the AstraZeneca vaccine was analyzed as a separate binary outcome variable (yes/no). Prior influenza vaccination status (vaccinated vs. unvaccinated) was considered the main exposure variable. Generalized linear models (GLMs) with a binomial distribution and log link function were used to estimate adjusted risk ratios (RRs) and corresponding 95% confidence intervals (CIs) for the association between prior influenza vaccination and each reported adverse event. Adjusted models included age and sex as potential confounding variables.

The number of reported adverse events following the second dose of AstraZeneca vaccination was analyzed as count data, representing the frequency of adverse events reported by each participant (0, 1, 2, 3, 4, or ≥5 events). Generalized linear models (GLMs) with a Poisson distribution and log link function were applied to evaluate the association between explanatory variables and the frequency of reported adverse events. Results were expressed as adjusted incidence rate ratios (IRRs) with corresponding 95% confidence intervals (CIs). Subgroup analyses according to age group (≤40 and >40 years) and sex were performed to evaluate the consistency of the association between prior influenza vaccination and the frequency of reported adverse events following AstraZeneca vaccination. Separate models were fitted within each subgroup. In age-stratified analyses, models were adjusted for sex and comorbidities, while in sex-stratified analyses, models were adjusted for age and comorbidities.

Relative risk reduction (RRR), which is expressed as 1–RR, and number needed to vaccinate (NNV), which is expressed as 1/absolute risk reduction, indexes were calculated and reported for cases whose odds ratio was less than one (Barratt et al., 2004). Data were analyzed using STATA software version 14 (StataCorp, College Station, TX, USA).

## 3 Results

The total number of HWs in the target hospitals was 4000 people, of which 2200 and 1800 people had not received and received the influenza vaccine, respectively. 391 subjects from the not receiving and 389 subjects from the receiving group, all of whom received both doses of the AstraZeneca vaccine, were randomly selected (Fig. 1).

### 3.1 Demographic and descriptive characteristics of research units

A total of 780 people were included in the study, of which 427 (54.74%) were women and 353 (45.26%) were men. The average (standard deviation) age of the subjects was 36.8 (9.44) years. Other demographic information of the participants according to the seasonal influenza vaccine is shown in Table 1.

**Table 1:** Comparison of demographic characteristics of participants by seasonal influenza vaccine groups (received/not received).

Variable		Seasonal influenza vaccine		Total	P-value
		Not-received	Received		
Gender	Female	244 (62.4)	183 (47.0)	427	0.001
	Male	147 (37.6)	206 (53.0)	353	
Work ward					
Official		82 (21.0)	53 (13.6)	135	0.001
Emergency		145 (37.1)	153 (39.3)	298	
Non-infectious inpatient department		61 (15.6)	31 (8.0)	92	
Infectious inpatient department		71 (18.2)	125 (32.1)	196	
Diagnostic		32 (8.2)	27 (7.0)	59	
Education	Under diploma	40 (10.2)	33 (8.5)	73	0.001
	BA	77 (20.0)	43 (11.0)	120	
	BS	184 (47.0)	197 (50.6)	381	
	MSc	16 (4.0)	37 (9.5)	53	
	PhD	74 (18.9)	79 (20.3)	153	
Marital status	Single	141 (36.1)	126 (32.4)	267	0.280
	Married	250 (63.9)	263 (67.6)	513	
Chronic disease	No	386 (98.7)	376 (96.7)	762	0.055
	Yes	5 (1.3)	13 (3.3)	18	
age		36.0 ± 9.2	37.6 ± 9.5		0.018
Work Experience		9.2 ± 7.4	8.7 ± 7.2		0.307

*Comparison of age and work experience in 2 groups was done with independent *t*-test.

The frequency of adverse events following the second dose of AstraZeneca vaccine is presented in Table 2 according to influenza vaccination status. After adjustment for age, sex, and comorbidities, prior influenza vaccination was associated with a significantly lower risk of several adverse events, including fever (RR=0.71, 95% CI: 0.60–0.83), chills (RR=0.75, 95% CI: 0.57–0.99), body pain (RR=0.76, 95% CI: 0.69–0.83), gastrointestinal symptoms (RR=0.37, 95% CI: 0.25–0.53), and fatigue (RR=0.70, 95% CI: 0.62–0.78), compared with the unvaccinated group. No statistically significant associations were observed for runny nose, eye irritation, headache, nausea, loss of smell, sore throat, or shortness of breath (Table 2).

**Table 2:** Association between prior influenza vaccination and adverse events following the second dose of AstraZeneca vaccine.

Side Effects: No/Yes		Unvaccinated n(%)	Influenza vaccinated n(%)	Adjusted RR* (95% CI)	RRR (%)	NNV
Runny nose	No	333 (85.1)	338 (86.9)	1.00	-	-
	Yes	58 (14.9)	51 (13.1)	.88 (0.62, 1.25)	12	58
Eye irritation	No	387 (98.9)	379 (97.4)	1.00	-	-
	Yes	4 (1.0)	10 (2.6)	2.5 (0.79, 7.9)		-
Fever	No	189 (48.3)	246 (63.2)	1.00	-	-
	Yes	202 (51.7)	143 (36.8)	.71 (0.60, 0.83)	29	7
Chills	No	300 (6.7)	321 (82.5)	1.00	-	-
	Yes	91 (23.3)	68 (17.5)	.75 (0.57, 0.99)	25	17
Loss of smell	No	382 (97.7)	380 (97.7)	1.00	-	-
	Yes	9 (2.3)	9 (2.3)	1 (0.4, 2.5)		-
Body pain	No	74 (18.9)	148 (38.0)	1.00	-	-
	Yes	317 (81.1)	241 (62.0)	.76 (0.69, 0.83)	24	5
Headache	No	258 (66.0)	238 (61.2)	1.00	-	-
	Yes	133 (34.0)	151 (38.8)	1.1 (0.94, 1.4)		-
Nausea	No	373 (95.4)	373 (95.9)	1.00	-	-
	Yes	18 (4.6)	16 (4.1)	.89 (0.50, 1.72)	11	-
Gastrointestinal symptoms	No	299 (6.5)	355 (91.3)	1.00	-	-
	Yes	92 (23.5)	34 (8.7)	.37 (0.25, 0.53)	63	7
Sore throat	No	376 (96.2)	360 (92.5)	1.00	-	-
	Yes	15 (3.8)	29 (7.5)	1.9 (1.06, 3.6)		-
Shortness of breath	No	387 (99.0)	380 (97.7)	1.00	-	-
	Yes	4 (1.0)	9 (2.3)	2.3 (0.70, 7.2)		-
Fatigue	No	94 (24.0)	182 (46.8)	1.00	-	-
	Yes	297 (76.0)	207 (53.2)	.7 (0.62, 0.78)	30	5

RRR: Relative Risk Reduction, NNV: Number Need to Vaccine, RR: risk ratio, CI: confidence interval,.

⁎ adjusted for age and gender, the outcome variable is “Adverse Events/Side Effects”, the reference group (RR = 1.00) is set as the “unvaccinated group.”

The highest efficacy (the potential protective effect) of influenza vaccine according to the RRR index was related to gastrointestinal side effects at 63%. It means that 63% of gastrointestinal symptoms are reduced in the receiving influenza vaccine group compared with the control group. Also, the calculation of the NNV index showed that for preventing one additional gastrointestinal symptoms related to AstraZeneca vaccine at least seven subjects need to be vaccinated against influenza (Table 2).

Table 3 presents subgroup analyses evaluating the association between prior influenza vaccination and the frequency of reported adverse events following AstraZeneca vaccination. The outcome variable in Poisson regression models was the number of adverse events reported by each participant. Among participants aged ≤40 years, prior influenza vaccination was associated with a lower frequency of reported adverse events (adjusted IRR=0.81, 95% CI: 0.73–0.89). A similar inverse association was observed among participants aged >40 years, although the association was weaker and did not remain statistically significant (adjusted IRR=0.92, 95% CI: 0.78–1.10).

**Table 3:** Subgroup analyses of the association between influenza vaccination and the number of reported adverse events per participant following AstraZeneca vaccination.

	Frequency of side effects (no (%))						Adjusted IRR	P-value
	0	1	2	3	4	5≤		
Total	42 (5.5)	108 (13.8)	182 (23.3)	210 (26.9)	140 (17.9)	98 (12.6)	-	
Age group								
≤ 40 year	20 (47.6)	41 (38.0)	106 (58.2)	152 (72.4)	108 (77.1)	94 (95.9)	0.81 (0.73, 0.89)	0.001
>40 year	22 (52.4)	67 (62.0)	76 (41.8)	58 (27.6)	32 (22.9)	4 (4.1)	0.92 (0.78, 1.10)	0.365
Gender group								
Female	17 (40.5)	42 (38.9)	84 (46.1)	117 (55.7)	93 (66.4)	74 (75.5)	0.85 (0.76, 0.96)	0.006
Male	25 (59.5)	66 (61.1)	98 (53.9)	93 (44.3)	47 (33.6)	24 (24.5)	0.81 (0.71, 0.93)	0.002
Influenza vaccination status								
Unvaccinated	8 (19.1)	41 (38.0)	87 (47.8)	105 (50.0)	79 (56.4)	71 (72.4)	Ref.	
Vaccinated	34 (80.9)	67 (62.0)	95 (52.2)	105 (50.0)	61 (43.6)	27 (27.6)	0.83 (0.76, 0.91)	0.001

IRR: Incidence rate ratio. The outcome variable was the number of reported adverse events per participant (0, 1, 2, 3, 4, or ≥5 events). IRRs were estimated using generalized linear models with Poisson distribution and log link function. In age-stratified analyses, models were adjusted for sex and comorbidities. In sex-stratified analyses, models were adjusted for age and comorbidities.

In sex-stratified analyses, prior influenza vaccination was associated with a lower frequency of reported adverse events among both female participants (adjusted IRR=0.85, 95% CI: 0.76–0.96) and male participants (adjusted IRR=0.81, 95% CI: 0.71–0.93). Overall, the direction of the associations was generally consistent across age and sex subgroups.

**Fig. 2:**
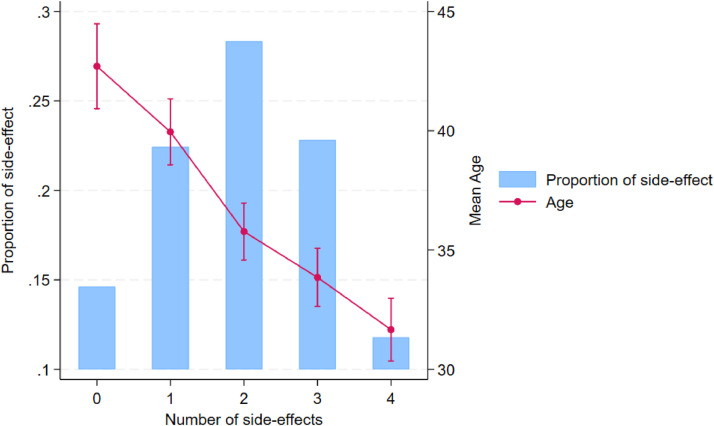
The relationship between the frequency of side effects of the AstraZeneca vaccine and the mean age of participants (Error bars indicate the 95% confidence intervals for the mean age).

**Fig. 3:**
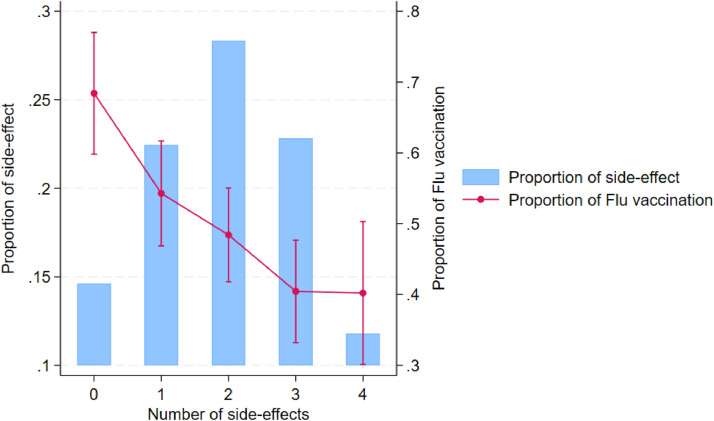
The relationship between the number of side effects of the AstraZeneca vaccine and influenza vaccine coverage *(*Error bars indicate the 95% confidence intervals for the mean age).

Fig. 2 shows that the number of side effects of the AstraZeneca vaccine decreases with age. In other words, the number of side effects is more common in younger people. Also, the relationship between influenza vaccine coverage and AstraZeneca vaccine side effects shows that the lower number of AstraZeneca vaccine side effects is associated with the higher coverage of the influenza vaccine (Fig. 3).

## 4 Discussion

This study investigated the association between prior seasonal influenza vaccination and the frequency of adverse events following AstraZeneca COVID-19 vaccination among healthcare workers. To our knowledge, limited studies have evaluated this specific association, and only one comparable study was identified in the available literature (Rzymski et al., 2022).

The most common side effects of the AstraZeneca vaccine observed in the present study were body pain, fatigue, fever, and chills. In addition, the majority of healthcare workers (95.5%) reported experiencing at least one side effect following vaccination, which is consistent with findings from previous studies (Alemayehu et al., 2022; Riad et al., 2021). A cross-sectional study conducted by Raid et al. in Germany and the Czech Republic among healthcare workers reported that 96.4% of participants experienced at least one vaccine-related side effect. The most common systemic adverse events included fatigue, body pain, fever, chills, malaise, nausea, and headache (Riad et al., 2021). Similarly, Song et al. reported that injection site pain, fatigue, myalgia, and fever were the most frequently observed adverse events following the first dose of the AstraZeneca vaccine among healthcare personnel in South Korea (Song et al., 2021). Consistent with our findings, younger participants in these studies experienced a higher frequency of adverse events compared with older individuals.

In the present study, individuals aged <40 years reported a greater frequency of vaccine-related adverse events. Similar findings were reported in previous studies conducted in different populations (Alemayehu et al., 2022; Ramasamy et al., 2021; Riad et al., 2021; Song et al., 2021). This age-related pattern may reflect stronger innate and adaptive immune responses in younger adults, leading to increased reactogenicity following vaccination. In addition, our stratified analyses according to age and sex demonstrated that the direction of the observed associations remained generally consistent across subgroups.

Alemayehu et al. evaluated age and gender disparities in adverse effects following AstraZeneca vaccination in eastern Ethiopia and reported that approximately 96.3% of participants experienced at least one adverse effect (Alemayehu et al., 2022). Similar to our findings, adverse events were more frequently reported among younger individuals. The most commonly reported side effects included local pain, fatigue, fever, and headache (Alemayehu et al., 2022), which were also among the predominant symptoms observed in the present study.

In the present study, some specific adverse events, including eye irritation, headache, sore throat, and shortness of breath, were reported more frequently among participants who had previously received the influenza vaccine. However, the overall frequency of mild and severe reactogenicity outcomes was generally lower in influenza-vaccinated individuals compared with those who had not received the influenza vaccine.

These findings should be interpreted cautiously. Previous studies evaluating influenza vaccination have primarily focused on susceptibility to SARS-CoV-2 infection or severity of natural COVID-19 disease rather than adverse reactions following COVID-19 vaccination (Li et al., 2020; Marín-Hernández et al., 2021; Noale et al., 2020; Ragni et al., 2020). Therefore, direct comparison between these outcomes should be made carefully, as vaccine reactogenicity and clinical severity of natural infection represent biologically distinct endpoints.

One of the notable findings of the present study was the observed association between receiving the seasonal influenza vaccine and a lower frequency of some adverse events following AstraZeneca COVID-19 vaccination. While this finding is partially consistent with previous reports (Rzymski et al., 2022), the underlying biological mechanism remains uncertain. Several hypotheses may explain this association. Previous studies have suggested that influenza vaccination may induce nonspecific modulation of innate immune responses, sometimes referred to as “trained immunity,” which could potentially influence inflammatory responses following subsequent vaccinations. In addition, individuals who regularly receive influenza vaccination may differ in health-seeking behavior, baseline health status, or healthcare utilization patterns compared with unvaccinated individuals. Nevertheless, these explanations remain speculative, and the observational nature of the present study does not permit causal inference.

A cross-sectional study by Noale et al. involving 198,828 individuals demonstrated that higher influenza vaccination coverage was associated with reduced incidence and mortality of natural COVID-19 disease (Noale et al., 2020). Similarly, other studies reported lower rates of SARS-CoV-2 infection or milder disease among individuals who had received influenza vaccination (Li et al., 2020; Marín-Hernández et al., 2021; Ragni et al., 2020). However, unlike those studies, the present investigation specifically focused on vaccine-induced adverse events, which represent a separate clinical and immunological outcome.

The estimated relative risk reductions (RRRs) observed in this study ranged from 11% to 69%. Because no previous study has specifically evaluated the association between influenza vaccination and adverse events following AstraZeneca COVID-19 vaccination, direct comparison with existing literature is limited. However, a large meta-analysis reported an RRR of approximately 59% for laboratory-confirmed influenza infection among individuals receiving influenza vaccination (Demicheli et al., 2018). Although the clinical outcomes differ substantially from those investigated in the present study, these findings may provide a contextual framework for interpreting the magnitude of the observed associations.

In addition, the estimated number needed to vaccinate (NNV) ranged from 5 to 58 individuals to potentially prevent one vaccine-related adverse event. Previous influenza vaccine studies have reported NNV estimates of 71 and 29 for preventing one case of influenza disease and influenza-like illness (ILI), respectively (Demicheli et al., 2018). Nevertheless, these comparisons should be interpreted cautiously because the present study evaluated vaccine reactogenicity rather than prevention of infectious disease outcomes. Given the exploratory and observational nature of the current study, these estimates should be considered preliminary and require confirmation in larger prospective studies.

## 5 Limitations

Several limitations should be considered when interpreting the findings of this study. First, because adverse events were self-reported, recall bias may have occurred, particularly for mild symptoms. To minimize this issue, we attempted to reduce the interval between vaccination and data collection. Second, due to the observational cross-sectional design, causal relationships cannot be established. Third, although adjusted and stratified analyses were performed, residual confounding remains possible. Factors such as socioeconomic status, occupational stress, healthcare-seeking behavior, previous exposure to SARS-CoV-2, and underlying comorbidities may have differed between influenza-vaccinated and unvaccinated participants. In addition, some severe adverse events were relatively infrequent, which may have reduced statistical precision in subgroup analyses. Therefore, the findings should be interpreted as exploratory and hypothesis-generating.

## 6 Conclusion

In conclusion, prior influenza vaccination was associated with a lower frequency of reported adverse events following the second dose of the AstraZeneca COVID-19 vaccine. Similar associations were observed for several specific adverse events, as well as for the overall number of reported symptoms. Older age and male sex were also associated with a lower frequency of reported adverse events. However, given the observational nature of the study and the possibility of residual confounding, these findings should be interpreted cautiously. Further prospective and mechanistic studies are needed to clarify the biological and clinical significance of these associations.

## Ethics approval and consent to participate

Written informed consent was obtained from all participants in the study. The data of the participants were anonymous analyzed. This study was approved by the Ethics Committee of Kermanshah University of Medical Sciences (IR.KUMS.REC.1400.475). All methods were carried out by relevant guidelines and regulations. This study was conducted by the Declaration of Helsinki.

## Consent for publication

Not applicable.

## Availability of data and materials

The data are not publicly available due to privacy and ethical restrictions; however, the data are available from the corresponding author upon reasonable request.

## Funding

This study was funded by the deputy for research and development of 10.13039/501100005317Kermanshah University of Medical Sciences (No. 4000604). It had no role in data collection, analysis, interpretation, or writing of the manuscript.

## CRediT authorship contribution statement

**Masoud Sedaghat:** Writing – original draft, Methodology, Formal analysis, Conceptualization. **Farid Najafi:** Writing – review & editing, Methodology, Formal analysis. **Fatemeh Khosravi Shademani:** Writing – review & editing, Methodology, Formal analysis. **Shahab Rezaeian:** Writing – review & editing, Supervision, Methodology, Formal analysis, Conceptualization.

## Declaration of competing interest

The authors have no statement to declare and they have no conflicts of interest.
